# Predictors of remission from body dysmorphic disorder after internet-delivered cognitive behavior therapy: a machine learning approach

**DOI:** 10.1186/s12888-020-02655-4

**Published:** 2020-05-19

**Authors:** Oskar Flygare, Jesper Enander, Erik Andersson, Brjánn Ljótsson, Volen Z. Ivanov, David Mataix-Cols, Christian Rück

**Affiliations:** 1 Centre for Psychiatry Research, Department of Clinical Neuroscience, Karolinska University Hospital, Karolinska Institutet, M46, SE-141 86 Huddinge, Sweden; 2 Stockholm Health Care Services, Region Stockholm, Stockholm, Sweden; 3 Division of Psychology, Department of Clinical Neuroscience, Karolinska Institutet, Solna, Sweden; 4 CAP Research Centre, Stockholm Health Care Services, Region Stockholm, Stockholm, Sweden

**Keywords:** Body dysmorphic disorder, Cognitive behaviour therapy, Internet, Predictor, Machine learning

## Abstract

**Background:**

Previous attempts to identify predictors of treatment outcomes in body dysmorphic disorder (BDD) have yielded inconsistent findings. One way to increase precision and clinical utility could be to use machine learning methods, which can incorporate multiple non-linear associations in prediction models.

**Methods:**

This study used a random forests machine learning approach to test if it is possible to reliably predict remission from BDD in a sample of 88 individuals that had received internet-delivered cognitive behavioral therapy for BDD. The random forest models were compared to traditional logistic regression analyses.

**Results:**

Random forests correctly identified 78% of participants as remitters or non-remitters at post-treatment. The accuracy of prediction was lower in subsequent follow-ups (68, 66 and 61% correctly classified at 3-, 12- and 24-month follow-ups, respectively). Depressive symptoms, treatment credibility, working alliance, and initial severity of BDD were among the most important predictors at the beginning of treatment. By contrast, the logistic regression models did not identify consistent and strong predictors of remission from BDD.

**Conclusions:**

The results provide initial support for the clinical utility of machine learning approaches in the prediction of outcomes of patients with BDD.

**Trial registration:**

ClinicalTrials.gov ID: NCT02010619.

## Background

Body dysmorphic disorder (BDD) has an estimated prevalence of about 2% [1, 2] in the general population, and is characterized by a distressing and impairing preoccupation with perceived defects or flaws in physical appearance that are not noticeable, or only appear slight to others [3]. Thoughts about the disliked body parts are perceived as intrusive, difficult to control and time consuming, leading to a disproportionate preoccupation [4]. Insight about the perceived defects varies on a continuum from fair to delusional, but is typically poor [5, 6]. Another hallmark of BDD is the presence of repetitive behaviours (such as compulsive mirror gazing, excessive grooming or camouflaging of disliked body areas) in an attempt to hide or control the perceived defects [3]. BDD is associated with occupational impairment [7], suicidality [8], and reduced quality of life [9].

Evidence-based treatments for BDD include selective serotonin re-uptake inhibitors (SSRIs) and cognitive behavioural therapy (CBT). Both treatment modalities are recommended in clinical guidelines [10] (published in 2005 and about to be updated) but the current evidence does not allow clinicians to decide which patients should be offered which treatment or whether they should be offered both. Furthermore, while these treatments are efficacious, outcomes are relatively modest, with few patients achieving remission and many requiring long-term treatment. For example, a recent meta-analysis of CBT studies indicated that only 40—54% of patients enrolling in these trials were classified as treatment responders [11], which is lower than CBT for obsessive-compulsive disorder where response rates are 62–68% [12].

Identification of reliable predictors of treatment outcome would potentially help guide clinical decision making and reduce the number of treatment failures. In a scenario where the prediction model indicates a low probability of treatment success, a patient could be offered an alternative or adjunct treatment option, or receive additional support from their therapist. Co-morbid depression, high baseline severity of BDD symptoms, and longer duration of BDD, have been found to predict outcomes of CBT for BDD in some studies [13, 14], but a recent meta-analysis on CBT for BDD [11] did not find any predictors that consistently predicted outcomes across studies. One possible explanation for these previous mixed results could be that previous studies have used stepwise regression models. Stepwise regression models are prone to bias in regression coefficients [15] and are therefore not recommended for prediction in psychiatric research. Despite these limitations, stepwise regression is the most common statistical technique used for prediction (but see [16] for a comprehensive review and guide to prediction in psychiatry).

One way to increase statistical precision and improve clinical utility could be to use machine learning techniques [17]. Machine learning uses an iterative approach where the statistical model is evaluated in small subsets of the available data to maximize predictive power. One specific machine learning technique is the random forest algorithm, which can detect and aggregate weak predictors in its statistical model without assumptions of normal distribution [18, 19]. Such techniques can make use of more of the available data compared to regression models, and are therefore ideal when there are many potential predictors relative to the number of participants. Random forests have been used in the prediction of time in remission from obsessive-compulsive disorder with an error rate of 25% [20]. Researchers have evaluated four different machine learning methods in the prediction of response to Internet-based CBT (ICBT) for obsessive-compulsive disorder in children and adolescents, with 75 to 83% accuracy (the proportion of correct classifications) [21]. Similarly, a combined model of random forest and elastic net algorithms consistently outperformed a traditional linear regression model when predicting results after ICBT for depression [22]. These initial results are encouraging and machine learning algorithms such as random forests are yet to be applied to outcome prediction in BDD.

This study reports on a secondary analysis of data from a recent clinical trial investigating the efficacy of ICBT for adults with BDD [23, 24]. We used random forests machine learning approaches to identify predictors of remission from BDD immediately after treatment and at various follow-up points. For comparison purposes, we also employed traditional logistic regression models. Remission status was chosen as the outcome of interest because it can be assessed in a standardised way and represents a large improvement in well-being that is meaningful for patients. Candidate predictor variables included demographic characteristics, clinical characteristics of BDD and co-occuring disorders, and treatment-related characteristics.

## Methods

### Study design

We used data from a previously published RCT [23, 24], registered at ClinicalTrials.gov (no. NCT02010619), in which 94 participants with a primary diagnosis of BDD were randomized to either BDD-NET (*n* = 47) or online supportive therapy (n = 47) for 12 weeks. Participants who had received supportive therapy were offered BDD-NET after the controlled three month follow-up, which 41/47 (87%) participants accepted. Predictor analyses were thus conducted on the entire sample that received BDD-NET (*n* = 88). Outcome data was gathered at post-treatment, 3-month follow-up, 12-month follow-up, and 24 months after treatment with BDD-NET. For a detailed description of recruitment, participants and procedures of the trial, please refer to the original publications [23, 24].

### Participants

All participants had a principal diagnosis of BDD according to the DSM-5, and a score of at least 20 on the modified Yale-Brown Obsessive-Compulsive Scale (BDD-YBOCS) [25]. Exclusion criteria were psychotropic medication changes within 2 months prior to enrolment, completion of CBT for BDD within the last 12 months, current substance dependence, a history of bipolar disorder or psychosis, acute suicidal ideation, a severe personality disorder that could jeopardize treatment participation (e.g., borderline personality disorder with self-harm), or concurrent psychological treatment.

### Predicting variables

Available predictor variables included demographic characteristics, clinical characteristics of BDD and co-occuring disorders, and treatment-related characteristics.

Potential *demographic predictors* were gender, age, level of education, occupational status, marital status, and whether participants had children or not.

*Clinical characteristics* were assessed by both clinicians and participants themselves. Clinicians diagnosed BDD using the structured clinical interview for DSM-IV axis I disorders with an added question about repetitive behaviors to reflect updates to the diagnostic criteria of BDD in DSM-5 (SCID-I), and used the Mini International Diagnostic Interview (MINI [26];) to determine whether comorbid conditions were present. Clinicians also assessed BDD symptom severity using the BDD-YBOCS [25], level of insight (good, poor, or delusional), clinical severity using the clinical global impression scale (CGI [27];), and overall level of functioning (GAF [3];). Participants self-reported depressive symptoms on the Montgomery Åsberg Depression Rating Scale (MADRS-S [28];), quality of life on the EuroQol 5-dimensions (EQ-5D [29];), body areas of concern, duration of BDD, medication with antidepressants, whether they had received previous psychological treatment for BDD, had been in contact with secondary psychiatric care (for any reason), or had undergone previous plastic surgery.

*Therapy-related predictors* included participant-rated treatment credibility and expectancy of improvement with the Credibility Scale (C-scale [30];) at week 2 post-baseline, and working alliance (i.e. agreement on goals, experiencing the therapist as supportive) according to the working alliance inventory short-revised (WAI-SR [31];) at week 2 in treatment. At the end of treatment, participants reported the overall time spent on the treatment. The treating therapists reported the number of completed modules.

### Definition of remission

Based on international expert consensus criteria, remission was defined as no longer fulfilling DSM-5 diagnostic criteria for BDD at the follow-up assessment [32].

### Procedure

Interested individuals registered for the study online and answered a screening questionnaire (demographic variables and clinical characteristics) and MADRS-S via the online platform. Trained assessors then conducted telephone interviews to establish the diagnosis of BDD using the SCID-I and co-morbid conditions using MINI, and rated BDD symptom severity (BDD-YBOCS), clinical severity on the clinical global impression scale (CGI), global assessment of functioning (GAF), and criteria for inclusion and exclusion before enrolment in treatment. At week 2 in treatment, participants rated treatment credibility (C-scale) and working alliance (WAI-SR). At post-treatment and follow-up (3, 12, and 24 months after treatment with BDD-NET) trained assessors conducted telephone assessments similar to the baseline assessment. Self-reported measures (MADRS-S, EQ-5D) were administered using the online platform. Both telephone assessments and self-report measures via the internet have been found to be reliable and valid administration formats [33–35].

### Treatment

Therapist guided internet-based cognitive behavioural therapy for body dysmorphic disorder (BDD-NET) was delivered via a tailored online platform using a dedicated hospital server with encrypted traffic and a two-factor authentication (password and single-use code sent via SMS) to guarantee participant confidentiality. The treatment lasted 12-weeks, and none of the participants had any face-to-face contact with a therapist. BDD-NET consists of self-help texts and worksheets that are delivered in eight interactive modules, each devoted to a certain theme. The BDD-NET modules are: 1) psychoeducation, 2) a CBT model for BDD, 3) cognitive restructuring, 4–5) exposure and response prevention and its application, 6) values-based behavior change, 7) difficulties encountered during treatment, and 8) relapse prevention plan. Throughout the treatment, the participant had unlimited access to an identified therapist that could be contacted at any time through the platform’s built in message system. The BDD-NET treatment protocol has been validated in a pilot trial [36], and was shown to be efficacious in the randomized controlled trial on which the current study is based [23], with gains maintained at 2-year follow-up [24].

### Statistical analyses

The random forest classification model was estimated using 10-fold cross-validation with 10 repeats to increase model stability [37]. In 10-fold cross-validation, the model is trained on 90% of the data and evaluated on the remaining 10%. The procedure is repeated for each of the 10 folds and the receiver operating characteristic (sensitivity and specificity) are averaged across repetitions. Random forest classifications were fitted using a maximum of 500 trees and varying the number of predictors used for each split. In random forests, multiple decision trees are created using a random subset of predictors, where the predictor—and its optimal cut-point—that minimizes classification error is chosen at each split. The final model was selected based on the receiver operating characteristic and evaluated by comparing the probability of remission from BDD estimated from the model to the actual remission or non-remission for each patient. Variable importance in the random forest models was determined by their effect on the overall accuracy of the model; more important variables resulted in a bigger accuracy gain when included. The effects of the eight most important individual predictors was investigated using partial dependence plots, where the association between different values of the predictor and the probability of remission is plotted while holding all other predictors constant at their mean value.

For the logistic regression analyses, we first fitted each predictor variable separately in univariate analyses. Predictors with *p* < .05 were then included in a multiple logistic regression, where we report odds ratios of remission versus non-remission for predictors with *p* < .05. An odds ratio below 1 means lower odds of remission, while an odds ratio higher than 1 indicates higher odds of remission. This procedure is similar to previous work in internet-delivered cognitive behavioural therapy [38].

Predictor variables with high collinearity and with near zero variance were removed prior to modelling, because including them would have made the logistic regression models unstable and provided an unfair advantage for the random forest algorithms. Included variables are marked with an asterisk in Table 1. Missing data was imputed using random forests in the *missForest* package [39]. Scripts for data preparation and statistical analyses, as well as detailed results from the random forest and regression analyses, are available on the Open Science Framework (https://osf.io/fu5sj/). We used R version 3.5.3 [40] for all statistical analyses.

**Table 1:** Characteristics of study participants used for prediction

**Variable**	**No. (%)**
**Sociodemographic variables**	
*Gender*	
Female^*^	74 (84%)
Male^*^	14 (16%)
Age, mean (SD)^*^	32.48 (11.62)
*Household structure*	
Married^*^	14 (16%)
Have children^*^	33 (38%)
*Level of education*	
Primary school^*^	11 (12%)
Secondary school^*^	50 (57%)
University degree^*^	26 (30%)
Doctorate	1 (1%)
*Occupational status*	
Working^*^	49 (56%)
Student^*^	22 (25%)
Unemployed^*^	13 (15%)
Disability pension	1 (1%)
Retired	3 (3%)
**Clinical variables**	
Years with BDD, mean (SD)^*^	18.83 (13.27)
*Insight*	
Good^*^	46 (52%)
Poor^*^	34 (39%)
Delusional^*^	8 (9%)
No. body areas of concern, mean (SD)	7.67 (4.68)
*Comorbidity*	
Current depressive episode^*^	44 (50%)
Panic disorder	3 (3%)
Social anxiety disorder^*^	25 (28%)
Obsessive-compulsive disorder^*^	8 (9%)
Generalized anxiety disorder^*^	15 (17%)
Bulimia nervosa	9 (10%)
ADHD	2 (2%)
*Medication*	
SSRI^*^	12 (14%)
SNRI	2 (2%)
Other antidepressant^*^	7 (8%)
*Previous treatment*	
Cognitive behavioral therapy for BDD^*^	10 (11%)
Other psychological treatment^*^	53 (60%)
Previous contact with psychiatry^*^	54 (61%)
Plastic surgery^*^	21 (24%)
No. of plastic surgeries, mean (SD)^*^	0.56 (1.30)
**Baseline measurements**	
*CGI-Severity*	
2 - Borderline mentally ill	3 (3%)
3 - Mildly ill^*^	13 (15%)
4 - Moderately ill^*^	47 (53%)
5 - Markedly ill^*^	21 (24%)
6 - Severely ill	3 (3%)
7 - Among the most extremely ill patients	1 (1%)
GAF, mean (SD)^*^	56.24 (6.67)
BDD-YBOCS, mean (SD)^*^	27.74 (5.52)
EQ-5D, mean (SD)^*^	13.06 (3.55)
MADRS-S, mean (SD)^*^	18.92 (9.05)
**Treatment-related variables**	
Treatment credibility, mean (SD)^*^	31.59 (11.51)
Working alliance inventory, mean (SD)^*^	65.27 (13.15)
No. modules completed, mean (SD)^*^	6.25 (2.45)
*Time spent on treatment (per week)*	
1 h^*^	16 (18%)
2 h^*^	17 (19%)
3 h^*^	15 (17%)
4 h^*^	6 (7%)
5 h^*^	6 (7%)
6 h^*^	8 (9%)
7 h	3 (3%)
8 h	5 (6%)
9 h or more^*^	12 (14%)
**Outcome: Number of participants in remission**	
Post treatment	27 (31%)
3-month follow-up	37 (42%)
12-month follow-up	41 (47%)
24-month follow-up	53 (60%)

Abbreviations: *BDD* Body dysmorphic disorder, *ADHD* Attention deficit hyperactivity disorder, *SSRI* Selective serotonin reuptake inhibitor; *SNRI* Serotonin-norepinephrine reuptake inhibitor, *CGI* Clinical global impression scale, *GAF* Global assessment of functioning, *BDD-YBOCS* Yale-Brown obsessive compulsive scale modified for BDD, *EQ-5D* EuroQol 5-dimensions, *MADRS-S* Montgomery-Åsberg depression rating scale-self report

^*^Variable was used in prediction

## Results

The characteristics of the study participants at baseline and rates of remitted patients at post-treatment and follow-up are presented in Table 1. The sample was predominantly female, highly educated and working or studying. On average, they had had BDD symptoms for over 18 years and their BDD was in the moderately severe range at the time of participation. Slightly over half the sample had good insight. The most common current comorbid condition was depression (50% of participants). The majority of participants had had previous contact with psychiatric services, though few had received evidence-based treatment for BDD. The rates of participants in remission increased from 31% at post-treatment to 60% at 24-month follow-up (bottom of Table 1).

### Predictors at post-treatment

**Fig. 1:**
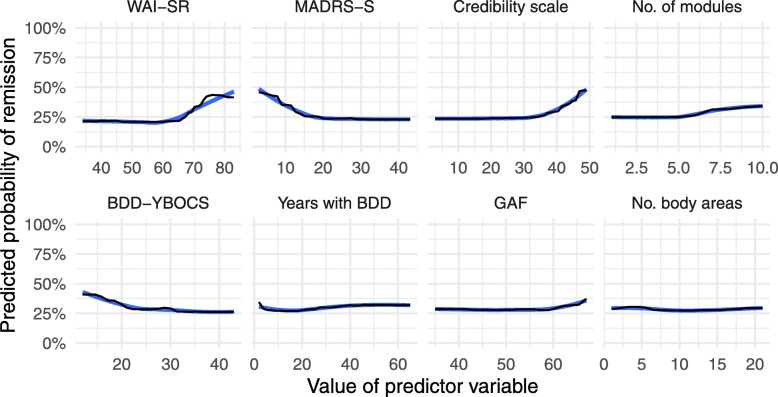
Partial dependence plots for remission status at post-treatment. Observed values (black) and LOESS-smoothing (blue) show the effect of each predictor variable when all other predictors are held constant at their mean value. Abbreviations: WAI-SR, Working alliance inventory short-revised; MADRS-S, Montgomery-Åsberg depression rating scale-self report; BDD-YBOCS, Yale-Brown obsessive compulsive scale modified for BDD; GAF, Global assessment of functioning

Twenty-seven patients or 31% of the sample were in remission at post-treatment. The random forest model correctly classified 78% of cases. Partial dependence plots for the eight most important predictors of remission status at post-treatment are shown in Fig. 1. Treatment credibility (Credibility scale) and working alliance (WAI-SR) rated at week 2 in treatment had a positive linear relationship with probability of remission, i.e. a higher score on these measures were associated with a higher probability of remission. Conversely, depressive symptoms (MADRS-S) and BDD symptoms (BDD-YBOCS) were negatively associated with probability of remission. The supplementary materials (https://osf.io/fu5sj/) include partial dependence plots for the most important predictors at follow-up.

The univariate logistic regression analyses identified 7 significant predictors, but only MADRS-S remained significant in the multivariate analysis (OR = 0.91 [95% CI 0.81–1], *p* = 0.041) after stepwise selection.

### Predictors at follow-up

Thirty-seven (42%) patients were in remission at the 3-month follow-up. The random forest model correctly classified 68% of cases. Treatment credibility, WAI-SR, MADRS-S and years with BDD were still among the most important variables. The univariate logistic regression analyses identified 8 predictive variables, but but only treatment credibility (C-scale) remained significant in the multivariate analysis (OR = 1.1 [95% CI 1.02–1.18], *p* = 0.019) after stepwise selection.

Fourty-one (47%) patients were in remission at the 12-month follow-up. The random forest model correctly classified 66% of cases. The most important predictor variable was treatment credibility. WAI-SR, MADRS-S, and BDD-YBOCS—all previously identified—were other important predictors. In the logistic regression, 8 variables passed the *p* < .05 threshold in univariate analyses. Treatment credibility (OR = 1.11 [1.04–1.19], *p* = 0.004) and spending at least 2 h per week on the treatment (OR = 10.99 [9.18–12.8], *p* = 0.009) remained statistically significant in the stepwise multivariate analysis.

Lastly, at the 24-month follow-up, 53 (60%) patients were in remission and the random forest model correctly classified 61% of cases. The most important predictor variables were MADRS-S, treatment credibility, number of body areas of concern, and BDD-YBOCS. In the logistic regression analyses, 6 variables reached statistical significance in the univariate model but only one remained statistically significant in the stepwise multivariate model: having children was associated with a higher probability of being in remission (OR = 3.59 [2.5–4.69], *p* = 0.022).

**Fig. 2:**
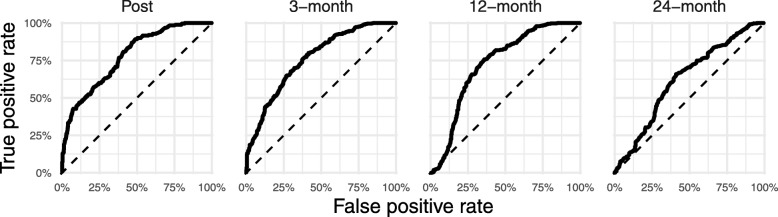
ROC-curves for random forest models. True positive and false positive rates at various thresholds

The random forest models were, in general, relatively more sensitive to detecting remitters than they were specific to correctly classifying non-remitters. The exception is at the 24-month follow-up, where the random forest model had high specificity at the expense of sensitivity (see Table 2). Figure 2 depicts model performance in terms of true and false positive rates at various thresholds for estimated probability of remission. The overall performance of the model can then be summarized as the area under the receiver operating characteristics curve (AUC) and compared to chance performance (dashed lines). AUC at post treatment was 0.78 and decreased over time (0.76, 0.73, and 0.63 at subsequent follow-ups), indicating that baseline predictor variables have less predictive power over time.

**Table 2:** Performance metrics of random forest models

Time	ROC	Sensitivity	Specificity
Post	0.78	0.92	0.43
3-month	0.78	0.82	0.50
12-month	0.73	0.71	0.66
24-month	0.64	0.23	0.88

Abbreviations: *ROC* Receiver operating characteristics

## Discussion

The results in this study indicated that a machine learning random forest algorithm could correctly detect 78% of participants as remitters or non-remitters at post-treatment. Although this accuracy was lower in the subsequent follow-ups, the model did still show acceptable predictive power compared to the traditional stepwise regression approach. The most important predictors were depressive symptoms, treatment credibility, working alliance, and initial severity of BDD. In general, the model was better able to detect true remitters and correctly classifying non-remitters. Although this was only a first ‘proof-of-concept’ study, the reults are encouraging and provide initial support for the use of machine learning algorithms to guide clinical decision making and approach personalized care for adults with BDD.

Depressive symptoms, measured using the MADRS-S, were consistently among the most important predictors in the random forest models. Previous trials of CBT for BDD using a questionnaire to measure depressive symptoms have found the same pattern [14], although a recent meta-analysis did not find that percentage of participants with diagnosed depression or antidepressant medication predicted results across trials [11]. Whether depression is measured by depressive symptoms on a severity scale or using a diagnosis of depression could explain the conflicting results; we found that symptoms—but not diagnosis—of depression was an important predictor, which is in line with previous findings [13, 14]. Our preliminary results point to the need for further treatment optimization for patients with co-morbid depressive symptoms, for example by adding specific modules targeting depressive symptoms in BDD-NET, or considering concurrent pharmacological treatment with antidepressants, for those that need it.

Our results also indicate that baseline severity and chronicity of BDD need to be taken into account. Baseline symptoms of BDD (BDD-YBOCS), number of body areas of concern, and years with BDD emerged as important predictors at every time-point. Initial severity and chronicity of BDD is an important part of the assessment prior to treatment selection, and the results are in line with [14], who found that BDD patients referred from secondary or tertiary care were less likely to respond to treatment. Similar observations were made in the early computerized CBT literature, e.g. in anxiety and mood disorders [41]. Level of insight into BDD was not an important predictor of remission, which is surprising given that insight is often cited as an important part of the assessment prior to CBT for BDD. However, individuals with low insight do not differ from individuals with adequate insight on most demographic and clinical characteristics [42]. Taken together, our results provide support for the idea of using ICBT for mild to moderate cases of BDD in a stepped-care model, where more severe cases receive other, more intensive interventions.

Treatment-related factors such as working alliance and treatment credibility may also be more important than demographic characteristics in the prediction of remission after ICBT for BDD. Both working alliance (WAI-SR) and treatment credibility (C-scale) emerged as important predictors at all time-points. Conversely, demographic characteristics were not important predictors of remission from BDD at any time-point. Similarly, the number of completed modules did not emerge as an important predictor at any time-point. It should be noted, however, that most participants completed the core modules that introduced exposure with response prevention, and may have continued to practice this technique despite not progressing to subsequent treatment modules. This indicates that success in ICBT for BDD is more likely to be determined by whether the user experiences the treatment as credible and the alliance with their therapist as positive, and that it is potentially helpful regardless of gender, age, family status, level of education or current occupational status.

Some limitations should be mentioned. First, the original randomized controlled trial consisted of self-referred patients with relatively good insight. Our sample differs slightly from previous trials of CBT for BDD, namely that participants in other clinical trials on BDD, on average, had higher levels of depressive symptoms [43, 44]. Second, although our sample size was relatively large, it was not large enough to divide into training and testing sub-samples that further decrease the possibility of false positive findings. Other measures were taken to reduce the risk of false positives, such as repeated cross-validation and averaging results across repeated models. Third, 41 of the 88 participants had received the supportive therapy control treatment before BDD-NET making the study sample more heterogeneous, as well as introducing potential additive effects after two bouts of treatment. However, only one individual was in remission after supportive therapy and of the 47 individuals initially randomised to supportive therapy 41 accepted BDD-NET as additional treatment [23]. Fourth, we do not know if these results will generalize to other treatment modalities for BDD, or whether integrating other sources of data, such as detailed platform usage, could potentially improve predictive accuracy.

Directions for future research include validating the use of machine learning algorithms in larger samples, developing algorithms that can guide clinical decision making (for example deciding between several available treatment options), and to integrate other kinds of data, e.g. genetic markers in the predictive models. Another suggestion for future research would be to do an experimental test of the clinical utility of machine learning prediction models. For example, patients could be randomized to either treatment as usual or a machine-learning-guided condition where patients at high-risk of non-remission are detected early using machine learning algorithms and offered additional treatment to maximize the chances of remission.

## Conclusions

The results of this proof-of-concept study show that machine learning algorithms such as random forests can be potentially useful to distinguish remitters from non-remitters after ICBT for BDD and provide initial support for the utility of machine learning techniques in personalized care in the treatment of BDD. Experimental designs testing these novel prediction models are needed.

## Data Availability

The datasets analysed during the current study are not publicly available since they contain sensitive personal identifying information and data sharing was not part of the written informed consent, but are available from the corresponding author on reasonable request. The scripts used for statistical analyses and additional materials are publicly available at (https://osf.io/fu5sj/).

## Abbreviations

AUC: Area under the receiver operating characteristics curve
BDD: Body dysmorphic disorder
BDD-NET: Therapist guided internet-based cognitive behavioural therapy for body dysmorphic disorder
BDD-YBOCS: Yale-Brown obsessive compulsive scale modified for BDD
CBT: Cognitive behaviour therapy
CGI: Clinical global impression scale
C-scale: Treatment credibility scale
DSM-5: Diagnostic and Statistical Manual of Mental Disorders, fifth edition
EQ-5D: EuroQol 5-dimensions
GAF: Global assessment of functioning
ICBT: Internet-based cognitive behaviour therapy
MADRS-S: Montgomery-Åsberg depression rating scale-self report
MINI: Mini International Diagnostic Interview
OCD: Obsessive-compulsive disorder
RCT: Randomised controlled trial
ROC: Receiver-operator characteristics
SSRI: Selective serotonin reuptake inhibitor
WAI-SR: Working alliance inventory short-revised
